# Comparison of the postoperative analgesic efficacy of the ultrasound-guided erector spinae plane block and intrathecal morphine in patients undergoing total abdominal hysterectomy under general anesthesia: a randomized controlled trial

**DOI:** 10.1007/s00540-025-03466-1

**Published:** 2025-03-06

**Authors:** Tarek Mohamed Ashoor, Ibrahim Mamdouh Esmat, Mohammad Abdalsalam Algendy, Noha Refaat Mohamed, Sahar Mohamed Talaat, Amal Hamed Rabie, Ahmed Mohammed Elsayed

**Affiliations:** 1 Department of Anesthesia, Intensive Care and Pain Management, Faculty of Medicine, Ain-Shams University, Cairo, Egypt; 2 Department of Clinical Pathology, Faculty of Medicine, Ain-Shams University, Cairo, Egypt

**Keywords:** General anesthesia, Total abdominal hysterectomy, Ultrasound, Erector spinae plane block, Intrathecal morphine, Postoperative, Pain, Analgesia

## Abstract

**Purpose:**

Total abdominal hysterectomy (TAH) is a common surgical procedure. Erector spinae plane block (ESPB) and intrathecal morphine (ITM) provide adequate postoperative (PO) analgesia. However, ITM side effects may limit its use. Researchers investigated the efficacy of bilateral ultrasound-guided ESPB on PO pain and analgesic consumption compared to ITM in the first 24 h following TAH under general anesthesia.

**Methods:**

120 patients premedicated with 3 mg intravenous granisetron were randomized into three equal groups: bilateral ultrasound-guided ESPB, ITM or control group. The primary outcome of this study was the time to first request for a rescue analgesic (tramadol).

**Results:**

Compared to the control group, the ESPB and ITM groups showed higher time to first request for a rescue analgesic and lower total tramadol consumption 24 h following surgery (*P* < 0.001) with significant differences between the ESPB and ITM groups (*P* < 0.001). The ITM group showed lower pain scores and lower readings of both serum glucose and cortisol levels compared to the other two groups 24 h after surgery (*P* < 0.001). The ITM group also had higher incidences of nausea and pruritus 24 h after surgery (*P* < 0.001)**.** The use of a single intrathecal injection of 0.3 mg morphine did not show any respiratory depression.

**Conclusion:**

0.3 mg intrathecal morphine was superior to erector spinae plane block for postoperative pain relief, 24 h after surgery, regarding attenuated stress response, lower pain scores at rest and on coughing and lower tramadol consumption.

**IRB:**

IRB 00006379//31-1-2022.

**Trial registration number:**

ClinicalTrials.gov Identifier: NCT05218733.

## Introduction

Patients undergoing total abdominal hysterectomy (TAH) suffer moderate to severe postoperative (PO) pain. Uncontrolled pain after TAH results in delayed postsurgical recovery, extended hospital stay, chronic pain, an increase in incidence of venous thrombosis and patient dissatisfaction [1]. The inadequate PO pain control in Africa due to poor PO pain assessment, the knowledge gap between medical workers, the patients’ lack of understanding and misconceptions, the scarcity of healthcare resources and the lack of pain relief medications remains a challenge for the anesthesiologists and acute pain service teams [2].

Accustomed analgesic regimens for major surgery depend on opioids to control postsurgical pain; however, the use of opioids may cause major adverse effects, e.g., sedation, respiratory depression (RD), postoperative nausea and vomiting (PONV), urinary retention and ileus, leading to delayed emergence from anesthesia and delayed discharge from hospital. Multimodal analgesic techniques optimize analgesia, eliminate the excessive use of opioids and decrease opioid-related side effects [3]. Abdominal field blocks are frequently used for pain control after abdominal surgeries such as laparotomies and appendicectomies and are considered as part of multimodal analgesia techniques [4].

The erector spinae plane block (ESPB) was first coined as a therapeutic approach for thoracic neuropathic pain. It is a peri-paravertebral regional anesthetic technique which was recorded as a suitable technique for pain management after surgery. In ESPB, the local anesthetic (LA) is deposited within the interfascial plane between the transverse process of the vertebra and erector spinae muscles. The ESPB safely allows for widespread of LA to multiple paravertebral spaces which blocks the ventral and dorsal rami resulting in blockade of both visceral and somatic pain [1, 5].

Intrathecal morphine injection (ITM), without LA, provided satisfactory PO analgesia and less PO rescue analgesia in patients undergoing major abdominal surgery under general anesthesia (GA) [6]. The analgesic technique, such as a single dose injection of ITM, has the potential to deliver the opioid directly into the cerebrospinal fluid (CSF), adjacent to the structures of the central nervous system where the opioid works [7]. Because of a small volume of distribution of the CSF and a slow diffusion, intrathecal hydrophilic opioids have a prolonged drug action in comparison with intravenous (IV) route of administration. A single drug injection technique of intrathecal hydrophilic opioids has an IV opioid-sparing effect, facilitates mobilization of patients and prevents peripheral vasodilation, which encourages restrictive fluid management. These benefits improve efficiency-related outcomes and enhance a faster recovery after abdominal surgery [8]. ITM develops a “ceiling” analgesic efficacy with the recommended intrathecal dose of 0.075–0.3 mg. Using larger doses may have deleterious events, mainly RD, without enhancing analgesia [9].

The stress response to surgery consists of two main components: neuroendocrine–metabolic response and inflammatory–immune response. Enhanced recovery after surgery (ERAS) protocols reduce the surgical stress response and improve PO recovery and PO outcomes [10]. ERAS protocols use multimodal analgesic regimens that include regional anesthetic techniques, systemic or neuraxial opioids to decrease the utilization of perioperative opioids and reduce their adverse effects to hasten the progress of patient recovery after surgery [11].

The research team performed this study on patients undergoing TAH under GA to compare the effects of the ultrasound (US)-guided bilateral single-injection ESPB with a single intrathecal injection of 0.3 mg morphine on PO pain and analgesic requirements in the first 24 h after surgery.

## Methods

### Ethics

After approval of the local ethical committee (IRB 00006379//31-1-2022), this study was registered prior to patient enrollment at ClinicalTrials.gov (NCT05218733) and adhered to standards of the Helsinki Declaration 2013. This clinical trial was performed between February 15, 2022 and September 20, 2023 at Ain-Shams University Maternity Hospital. Each patient signed a written informed consent before the operation.

### Study population

This clinical trial was conducted on 120 females, aged 40–55 years, body mass index (BMI) < 35 kg/m^2^, American Society of Anesthesiologists (ASA) I, II physical status, and listed for elective open TAH via Pfannenstiel incision under GA. Patients with hepatic or renal dysfunction, severe cardiovascular disease, coagulopathy, pulmonary function compromise, obstructive sleep apnea, diabetes mellitus or prediabetes, and pre-existing chronic pain were excluded. Patient’s refusal, local infection at site of block, known allergy to study drugs (bupivacaine or morphine), altered mental status and pre-existing steroid treatment were also causes of exclusion from this study.

### Randomization and blinding

After randomization using computer-generated random numbers kept in sealed opaque envelopes and based on PO pain management protocol with an allocation ratio of 1:1:1, patients were allocated to the ESPB group, ITM group or control group. An anesthesiology resident picked the envelope to know the patient`s assigned group. All patients received GA. By the end of surgery and before extubation, patients in the ESPB group obtained bilateral US-guided ESPB and sham block at the lumbar puncture site; patients in the ITM group obtained 0.3 mg of preservative-free morphine dissolved in 3 mL normal saline (NS) intrathecally and bilateral US-guided sham block at the ESPB puncture sites and patients in the control group obtained a bilateral US-guided sham block at the ESPB puncture sites and a sham block at the lumbar puncture site. Both the ESPB and ITM groups were the intervention groups. US-guided ESPB was performed by experienced anesthesiologists who had no additional role in this clinical investigation. Every sham block was conducted using 2 mL NS solution of a subcutaneous injection at the corresponding block site. Anesthesiology residents, blinded to patient allocation, evaluated and recorded the clinical trial outcomes. The procedures were performed by the same experienced gynecologic surgeons’ team. Patients and surgeons were also blinded to allocation of treatment groups.

### Study non-dependent protocol

Following the fulfillment of routine preoperative evaluation, patients were taught about the visual analog scale (VAS) (0–10 cm) to rate nausea and pain at rest and with cough [12]. Nausea was rated from 0 = no nausea to 10 = the worst nausea imaginable, and pain was rated from 0 = no pain to 10 = the worst pain imaginable. Patients received 150 mg ranitidine tablet the night before surgery and abstained from solid food for 6 h prior to surgery. Patients were also premedicated with 3 mg IV granisetron, and antibiotic prophylaxis after establishment of an intravenous (IV) line. A baseline reading of vital data was obtained for every patient after applying the standard operating room monitors.

Induction of GA was established with fentanyl 1.5 µg/kg and propofol 1.5 mg/kg, followed by rocuronium 0.6 mg/kg to facilitate orotracheal intubation. Following intubation, a Foley catheter was inserted for measurement of urine output and remained in place for 24 h. Anesthesia was maintained with 1–1.5% isoflurane with 50% oxygen in air and a top*-*up of muscle relaxant was provided on demand. All patients were mechanically ventilated to keep the EtCO_2_ between 35 and 40 mmHg. Additional boluses of fentanyl (0.5 ug/kg) were given if heart rate (HR) or mean arterial pressure (MAP) increased more than 20% from baseline values and the total intraoperative fentanyl doses were documented. 1 g IV acetaminophen was administered for all patients 15 min before the end of surgery and every 6 h till 24 h following surgery. By the end of surgery, every patient was laterally positioned and underwent an ESPB, spinal anesthesia or sham block according to the patient`s group allocation, followed by placing in the supine position again. The inhalational anesthetic was turned off and the residual neuromuscular paralysis was reversed by neostigmine (0.05 mg/kg) and atropine (0.01 mg/kg). After extubation, every patient was sent to the post-anesthesia care unit (PACU). The hemodynamic variables** (**HR and MAP) of patients were documented at T0: before induction of GA; T1: mean intraoperative changes of both HR and MBP before the procedure (ESPB, ITM or sham block); T2: 20 min after performing the block; T3, T4, T5, T6 and T7 which matched 2, 6, 12, 18 and 24 h postsurgery, respectively. VAS pain scores at rest and on coughing were assessed at 30 min, 2, 6, 12, 18 and 24 h after surgery. The PO level of sedation of every patient was appraised using the Richmond Agitation Sedation Scale (RASS) [13]. Patients were transferred to the gynecology ward when they got a modified Aldrete score ≥ 9.

### Study-dependent protocol

#### US-guided ESPB technique

**Fig. 1:**
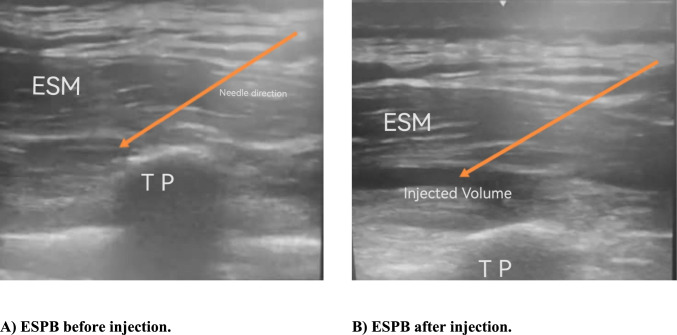
US pictures demonstrating the direction of the needle and anatomical landmarks during ESPB. [(**A**) ESPB before injection, (**B**) ESPB after injection)] (*ESM* erector spinae muscle, *ESPB* erector spinae plane block, *TP* transverse process, *US* ultrasound)

After skin sterilization with povidone-iodine solution, ESPB was achieved at the level of the spine of the ninth thoracic vertebra (T9). T9 was located by counting down from the spine of the seventh cervical vertebra. A high-frequency (5–13 MHz) linear US transducer (M‑turbo; Fujifilm SonoSite Inc., Bothell, Washington, USA) was placed vertically 3 cm lateral to the T9 spinous process. An 80 mm 22-gauge block needle (Stimuplex^®^ D, B-Braun, Germany) was placed using an in-plane approach. The needle tip was inserted into the fascial plane on the deep aspect of the erector spinae muscle (ESM). The correct location of the needle tip was verified by 2–3 ml of NS injectant for hydrodissection of the interfascial plane between the ESM and the transverse process. A total volume of 20 mL of 0.25% bupivacaine was injected through the needle. The same technique was repeated on the opposite side (Fig. 1**)**.

### ITM group

Morphine (10 mg/mL) was added to 100 ml saline dilution bag to achieve a concentration of 100 μgm/mL. The anesthesiologist assigned to perform the intrathecal injection drew 3 ml from the dilution bag in a 3 ml syringe. After skin sterilization with povidone-iodine solution, the dura was punctured at the L4–L5 or L5–S1 intervertebral space using a 25G Quincke spinal needle and the prepared 3 ml diluted morphine (0.3 mg) injected after confirming free flow of the CSF through the needle.

### Parameters and outcomes

HR and MAP were listed at different time points during the study. The time to perform the regional anesthesia technique was documented. All patients were treated with a rescue analgesia of 100 mg tramadol (ampoule) diluted in 100 ml of NS. This rescue dose was infused over 15 min for a VAS > 3 or patient`s request for analgesia and not exceeding 400 mg/day. The time to first request for a rescue analgesic and the total tramadol consumption/24 h after surgery were reported. Admitting more than one dose of rescue analgesic medication to the patient in the first hour after surgery was considered as a failed block which was recorded and omitted from the study results.

Recovery parameters including the length of PACU stay (min), time to unassisted ambulation (h), time to return of gastrointestinal function (passing flatus) (days) and hospital length of stay (LOS) (days) were listed.

The intervention related side effects including bradycardia, hypotension, PONV, pruritis, RD (respiratory rate < 12 breaths/min), LA toxicity and block technique adverse events (local site infection, hematoma formation and pneumothorax) were assessed. If patient experienced nausea at a VAS score > 7 or one emetic episode, she received 10 mg IV metoclopramide. Pruritus was appraised using a four-point scale [13] and patient with severe pruritus was treated with 45.5 mg IV pheniramine maleate (Avil^R^). Sedation, respiratory rate and oxygen saturation (SpO_2_) were monitored for 24 h after surgery. 0.4 mg IV naloxone was administered and supplemental oxygen by nasal cannula at 6 l/min if the patient had SpO2 ≤ 94% or RASS of − 4, or − 5. Patient satisfaction with PO pain control regimen was recorded at 24 h after surgery using a five-point Likert scale [14].

### Laboratory analysis

Three samples (3 mL each) of venous blood were collected from each patient included in this study under complete aseptic conditions; one hour before the operation as a baseline sample (8 a.m.) (H0), 2 h after the operation (H1) and 24 h postoperatively (H2) for assessment of serum glucose and serum cortisol. The blood was left to clot for 30 min in sterile dry vacutainers. The serum was then separated by centrifugation at 3500 rpm for 15 min, for the immediate assessment of serum glucose and serum cortisol. The analysis was done on COBAS e 402 and COBAS e 801 systems respectively supplied by Roche Diagnostics (GmbH, Sandhofer Strasse 116, D-68305 Mannheim).

The time to first rescue analgesic was the primary outcome measure of this study. Secondary outcome measures were VAS scores at rest and on coughing, the total tramadol consumption/24 h, hospital LOS, measurements of both serum glucose levels and morning serum cortisol levels, study-related adverse events and patient satisfaction.

### Statistical analysis

#### Power of the study

The results from a prior pilot study, conducted by our research team, showed that the time to first rescue analgesia in the ESPB, ITM and control groups was 19.7 ± 1.5, 21.0 ± 1.6 and 0.8 ± 0.2 h, respectively, setting the power at 80%, alpha error at 0.017 for the three groups’ comparisons [15]. Depending on PASS 11th release program for sample size calculation [16], a minimal sample size of 32 patients/group was required to get a statistically significant difference based on the minimum difference among groups. We recruited 40 patients/group for possible dropouts and to study other secondary outcomes.

#### Data analysis

Statistical Package for Social Sciences software version 28.0, IBM Corp., Chicago, USA, 2021, was used for data analysis. Qualitative variables were expressed as number and percentage and compared using Chi-square test as well as Fisher’s exact test for variables with small expected numbers. Quantitative variables were tested for normality using Shapiro–Wilk test, then expressed as mean ± standard deviation (SD) if normally distributed and compared using ANOVA (comparison between groups) and RMANOVA (comparison within groups) tests. If quantitative variables were not normally distributed, they were expressed as median (1st–3rd interquartile range) and compared using Kruskal–Wallis test (comparison between groups). Post hoc comparisons were done using Bonferroni test (comparison between groups, so homogenous groups had the same symbol “a, b or c”) and Dunnett’s test (comparison within groups, so times significantly different from T0 or H0 had symbol “⌂”). A *p* value < 0.050 was considered statistically significant.

## Results

**Fig. 2:**
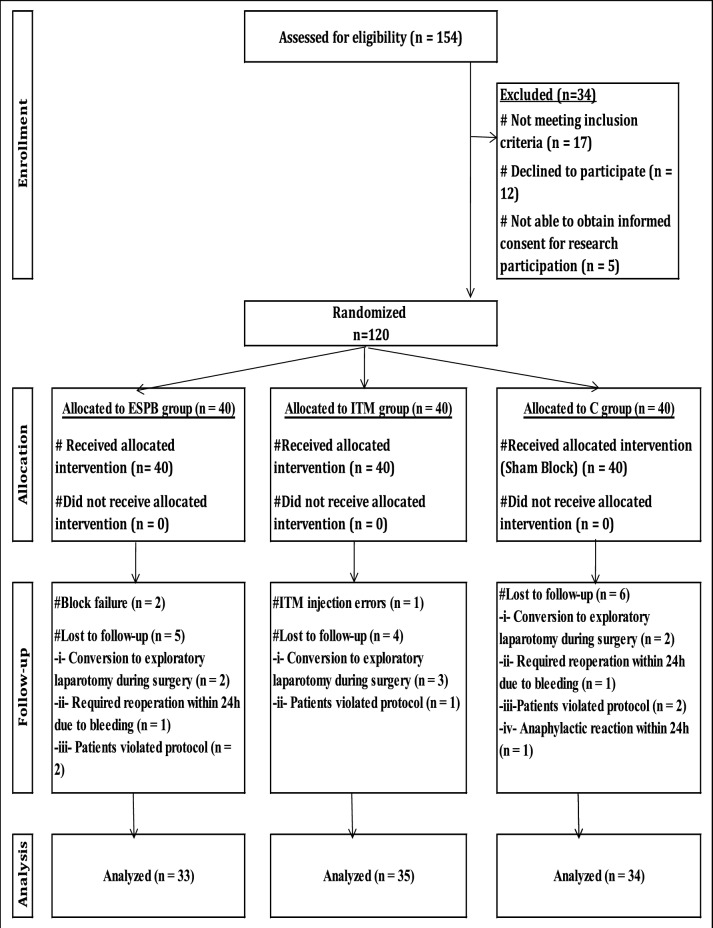
Flowchart of the study

154 patients were enrolled in this study. 34 patients were excluded and 120 patients were randomized to three equal groups. 18 patients were exempted from the final statistical analysis of this study due to different reasons (Fig. 2). The study participant demographic characteristics were comparable between groups (*P* > 0*.*05) (Table 1).

**Table 1:** Study participant demographic characteristics

Variables		ESPB group (Total = 33)	ITM group (Total = 35)	Control group (Total = 34)	*p* value
Age (years)		50.5 ± 2.5	49.5 ± 3.3	49.1 ± 2.9	*0.164
BMI (kg/ m^2^)		29.6 ± 2.3	29.5 ± 2.3	29.8 ± 2.3	*0.795
ASA	I	14 (42.4%)	15 (42.9%)	12 (35.3%)	†0.774
	II	19 (57.6%)	20 (57.1%)	22 (64.7%)	
Daily Smoker		4 (12.1%)	5 (14.3%)	8 (23.5%)	†0.409
Comorbidities					
Comorbidities (total)		7 (21.2%)	7 (20.0%)	9 (26.5%)	†0.793
Hypertension		7 (21.2%)	7 (20.0%)	9 (26.5%)	†0.793
Seizures		2 (6.1%)	0 (0.0%)	1 (2.9%)	‡0.209
Hypothyroidism		1 (3.0%)	2 (5.7%)	1 (2.9%)	‡0.999
Multiple comorbidities		3 (9.1%)	2 (5.7%)	2 (5.9%)	‡0.793
Surgical diagnosis					
Endometrial carcinoma		10 (30.3%)	11 (31.4%)	9 (26.5%)	‡0.995
Adnexal mass		6 (18.2%)	5 (14.3%)	7 (20.6%)	
Fibroid uterus		15 (45.5%)	16 (45.7%)	16 (47.1%)	
Dysfunctional uterine bleeding (DUB)		2 (6.1%)	3 (8.6%)	2 (5.9%)	
Duration of operation (min)		122.9 ± 9.0	120.2 ± 9.4	122.4 ± 10.0	*0.467
Intraoperative fentanyl consumption (µg)		224.3 ± 13.1	226.7 ± 10.9	222.4 ± 9.8	*0.286
Blood loss (ml)		523.9 ± 26.0	516.9 ± 43.1	509.2 ± 36.9	*0.255
Intraoperative fluid (ml)		1495.8 ± 119.3	1506.0 ± 123.4	1484.7 ± 88.4	*0.731

Data are presented as mean ± SD or number (%). BMI: body mass index. ASA: American Society of Anesthesiologists. *ANOVA test. †Chi-square test. ‡Fisher’s exact test

**Fig. 3:**
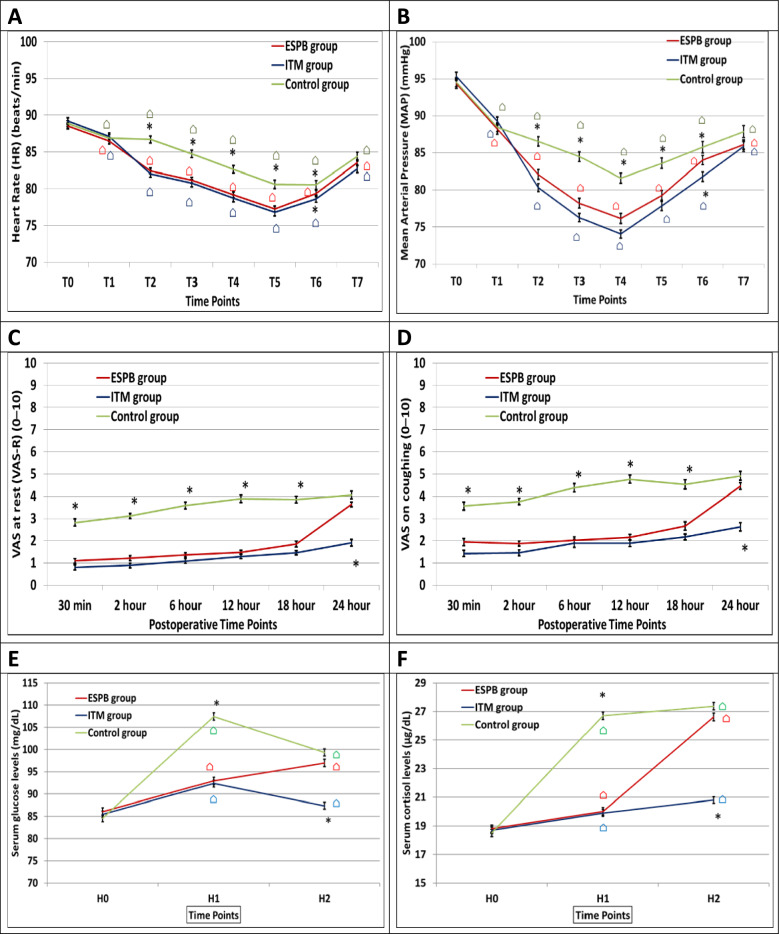
Hemodynamic variables changes, visual analog scale (VAS) scores and surgery-related stress response between study groups. **A** Heart rate changes, **B** mean arterial pressure changes, **C** VAS at rest, **D** VAS on coughing, **E** serum glucose levels, **F** serum cortisol levels (*significantly different group based on post hoc Bonferroni test following ANOVA test; times significantly different from T0 or H0 had the symbol “⌂” based on post hoc Dunnett test following RMANOVA test)

The investigators reported no statistically significant differences between groups (*P* > 0*.*05) regarding changes in HR and MAP values at the following time points; T0, T1, and T7 (Fig. 3). The ESPB and ITM groups exhibited lower HR and MAP (*P* < 0*.*001) values at T2, T3, T4, and T5 in comparison to the control group and the researchers found no statistically significant differences between the ESPB and ITM groups (*P* > 0*.*05) (Fig. 3). Compared to the control group, patients in the ITM group showed lower HR and MAP values at T6 (*P* = 0.034 for HR, & *P* < 0*.*001 for MAP) (Fig. 3). The HR and MAP readings (from T1 till T7) were significantly lower than T0 readings in all study groups (*P* < 0.001) (Fig. 3).

Patients of both ESPB and ITM groups had lower pain scores at rest and on coughing (*P* < 0.001) in comparison to the control group in the first 18 h postoperatively and the investigators found no statistically significant differences between the ESPB and ITM groups (*P* > 0*.*05) (Fig. 3). Nevertheless, patients in the ITM group showed lower pain scores at rest and on coughing (*P* < 0.001) compared to the other two groups 24 h after surgery and the researchers reported no statistically significant differences between the ESPB and control groups (*P* > 0*.*05) (Fig. 3).

The investigators found no statistically significant differences regarding baseline readings of serum glucose and cortisol levels between the groups at H0 (*P* > 0*.*05) (Fig. 3). There was a significant increase in serum glucose and cortisol levels in the ESPB, ITM and control groups at H1 and H2 compared to the baseline values (H0) (*P* < 0.001) (Fig. 3). The control group showed higher serum glucose and cortisol levels compared to both the ESPB and ITM (*P* < 0.001) groups at H1 and to the ITM group only at H2 (*P* < 0.001) (Fig. 3). The researchers found no significant differences between the ESPB and ITM groups at H1 (*P* > 0*.*05) and significant differences between the intervention groups at H2 (*P* < 0.001) (Fig. 3).

**Fig. 4:**
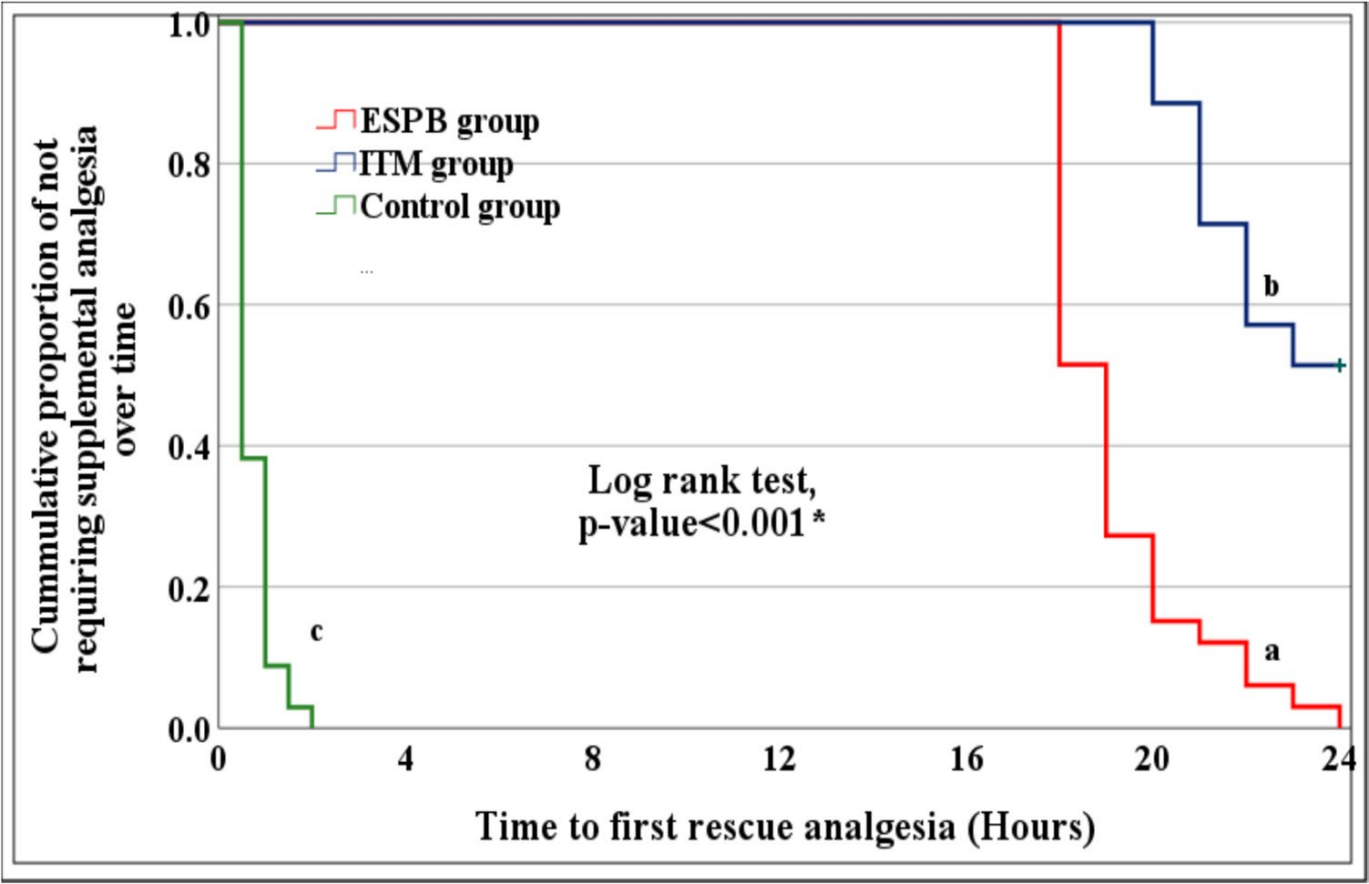
Kaplan–Meier curve for rate of requiring rescue analgesia between the study groups. (*significant, homogenous groups had the same symbol “a,b,c” based on post hoc Bonferroni test)

Compared to the control group, the time to perform the block and the time to first rescue analgesia were significantly prolonged in the ESPB and ITM (*P* < 0.001) groups and the investigators found significant differences between the ESPB and ITM groups (*P* < 0.001) (Table 2) (Fig. 4). Patients who required rescue analgesics, (number of doses and total tramadol consumption/24h) after surgery were higher in the control group than the intervention groups (*P* < 0.001) and the researchers found significant differences between the ESPB and ITM groups (*P* < 0.001) (Table 2) (Fig. 4).

**Table 2:** Clinical outcome differences between the study groups

Variables		ESPB group (total = 33)	ITM group (total = 35)	Control group (total = 34)	*p* value
Time to perform the block (min)		16.1 ± 0.9 a	8.1 ± 0.6 b	4.0 ± 0.6 c	* < 0.001§
Duration of anesthesia (min)		139.0 ± 9.1 a	128.3 ± 9.3 b	126.4 ± 10.1 b	* < 0.001§
Length of PACU stay (min)		26.2 ± 1.2 a	25.7 ± 1.1 a	33.4 ± 1.0 b	* < 0.001§
Number of patients who needed rescue analgesia		33 (100.0%) a	17 (48.6%) b	34 (100.0%) a	†** < 0.001**§
Time to first rescue analgesia (h) (in cases that needed rescue analgesia)		19.2 ± 1.6 a	21.3 ± 1.0 b	0.8 ± 0.4 c	* < 0.001§
Number of rescue doses (tramadol) given for each patient, 0–24 h (in cases that needed rescue analgesia)		1.5 ± 0.5 a	1.0 ± 0.0 b	2.9 ± 0.9 c	* < 0.001§
Total dose of rescue analgesia (tramadol), (mg) 0–24 h (in cases that needed rescue analgesia)		148.5 ± 50.8 a	100.0 ± 0.0 b	285.3 ± 85.7 c	* < 0.001§
Time to first ambulation (h)		12.6 ± 2.0 a	11.5 ± 2.0 a	15.9 ± 1.8 b	* < 0.001§
Time to return of gastrointestinal (GI) function (passing flatus) (h)		29.8 ± 5.1 a	27.7 ± 5.0 a	41.4 ± 4.6 b	* < 0.001§
Hospital length of stay (LOS) (days)		3.7 ± 0.6 a	3.4 ± 0.5 a	4.6 ± 0.8 b	* < 0.001§
Side effects					
Bradycardia		6 (18.2%)	4 (11.4%)	3 (8.8%)	‡0.495
Hypotension		6 (18.2%)	3 (8.6%)	2 (5.9%)	‡0.266
Nausea within 24 h		4 (12.1%) a	24 (68.6%) b	13 (38.2%) c	†** < 0.001**§
Nausea at a VAS score > 7		3 (9.1%)	9 (25.7%)	6 (17.6%)	†0.199
Vomiting		2 (6.1%)	7 (20.0%)	5 (14.7%)	‡0.262
Need for rescue antiemetic drug		3 (9.1%)	9 (25.7%)	6 (17.6%)	†0.199
Pruritus within 24 h		1 (3.0%) a	32 (91.4%) b	3 (8.8%) a	†** < 0.001**§
Severity of pruritus	None	32 (97.0%) a	3 (8.6%) b	31 (91.2%) a	‡** < 0.001**§
	Mild	1 (3.0%) a	26 (74.2%) b	3 (8.8%) a	
	Moderate	0 (0.0%) a	3 (8.6%) a	0 (0.0%) a	
	Severe	0 (0.0%) a	3 (8.6%) a	0 (0.0%) a	
Need for rescue antipruritic drug		0 (0.0%)	3 (8.6%)	0 (0.0%)	‡0.105
SpO_2_ (%)		98.3 ± 0.6	97.9 ± 1.0	98.4 ± 0.6	*0.061
Respiratory depression (RD)		0 (0.0%)	0 (0.0%)	0 (0.0%)	NA
Postdural puncture headache		0 (0.0%)	0 (0.0%)	0 (0.0%)	NA
Block technique adverse events		1 (3.0%)	0 (0.0%)	0 (0.0%)	‡0.324
LA toxicity		0 (0.0%)	0 (0.0%)	0 (0.0%)	NA
Constipation		2 (6.1%)	5 (14.3%)	1 (2.9%)	‡0.241
Patient satisfaction score (1–5) 24 h postoperatively		4.1 ± 0.6 a	4.4 ± 0.5 a	3.1 ± 0.8 b	* < 0.001§

Data are presented as mean ± SD or number (%). PACU: post-anesthesia care unit, SpO_2:_ oxygen saturation. *ANOVA test. †Chi-square test. ‡Fisher’s exact test Homogenous groups had the same symbol “a,b,c” based on post hoc Bonferroni test §Significant

The duration of anesthesia was highest in the ESPB group (*P* < 0.001) with comparable differences between the other two groups (*P* > 0*.*05) (Table 2). In comparison to the control group, the recovery parameters showed significant shorter time periods in the ESPB and ITM (*P* < 0.001) groups with no significant differences between the intervention groups (*P* > 0*.*05) (Table 2).

**Fig. 5:**
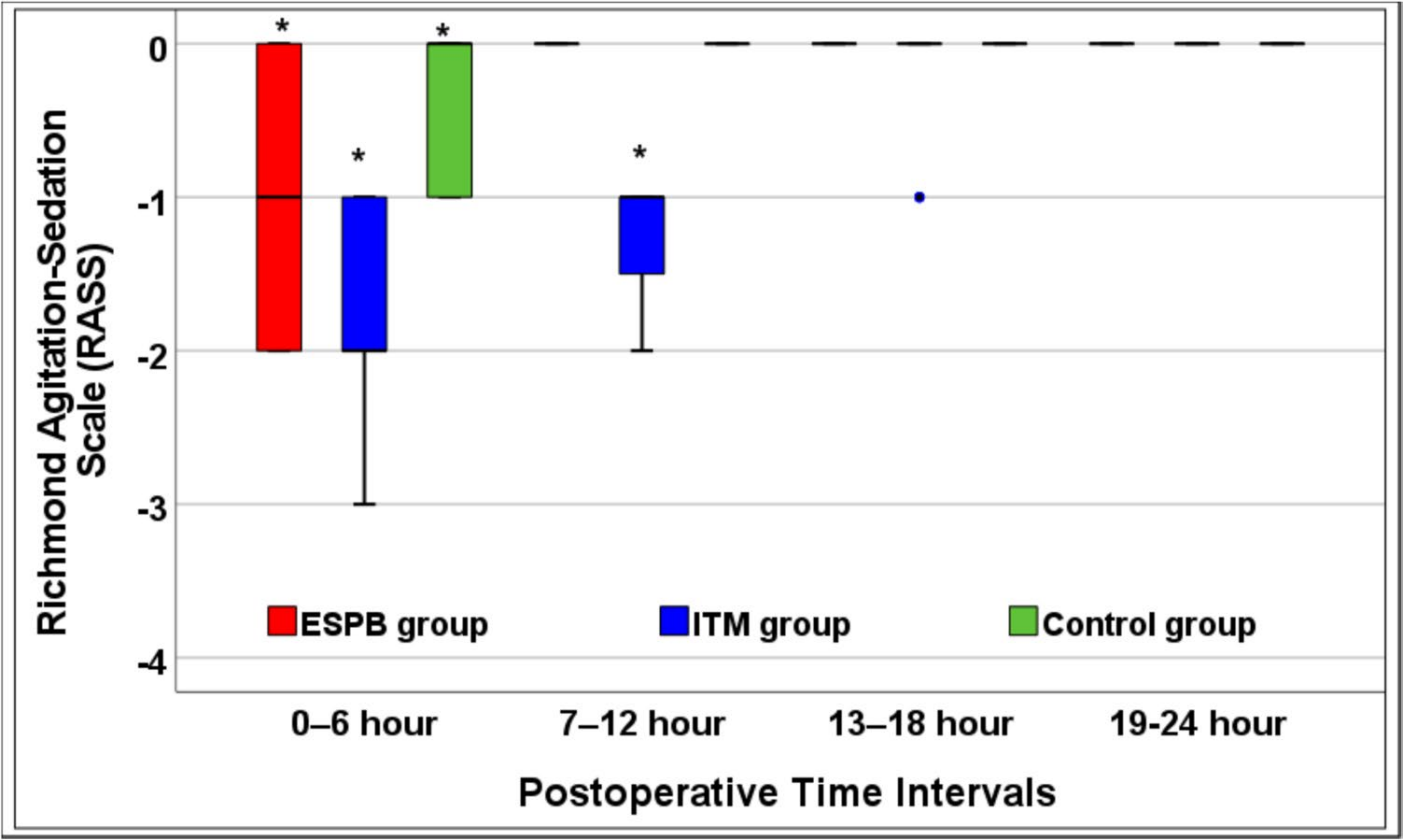
Richmond Agitation–Sedation Scale (RASS) between the study groups [*significantly different group based on post hoc Bonferroni test following Kruskal–Wallis test (●outlier reading)]

More patients in the ESPB and ITM groups exhibited significant PO sedation in comparison to the control group in the first 6 h postoperatively (*P* < 0.001) and the investigators found statistically significant differences between the ESPB and ITM groups (*P* < 0.001) (Fig. 5). The differences were comparable between the study groups in the next 18 h after surgery (*P* > 0*.*05) except at the 7–12 h interval postsurgery where patients in the ITM group showed higher levels of PO sedation compared to the other two groups (*P* < 0.001) (Fig. 5).

Compared to both ESPB and control groups, the ITM group had higher incidences of nausea (*P* < 0.001) and pruritus (*P* < 0.001) 24 h after surgery and the investigators found significant differences between the ESPB and control groups regarding the incidence of nausea (*P* < 0.001) (Table 2). Compared to the other two groups, most of the pruritus cases in the ITM group were of a mild form (*P* < 0.001) (Table 2) requiring no rescue antipruritic drug (*P* > *0.05*) (Table 2). Patient satisfaction score regarding postsurgical pain control was low in the control group compared to both the ESPB and ITM (*P* < 0.001) groups and the researchers found no significant differences between the ESPB and ITM groups (*P* > 0*.*05) (Table 2).

## Discussion

In this study, both ESPB and ITM demonstrated effective pain control, as evidenced by delayed requests for rescue analgesics, lower pain scores in the first 18 h postsurgery, reduced tramadol consumption and higher patient satisfaction compared to the control group. Patients in the ITM group experienced more nausea and pruritus with no cases of RD.

ESPB not only improves somatic pain (abdominal wall wound), but also provides visceral abdominal analgesia (uterus) [17]. In concordance with previous studies, patients who received US-guided ESPB had prolonged time to first rescue analgesia, reduced pain scores, decreased PO opioid consumption, early ambulation, shorter hospital stay and better patient satisfaction when compared with either conventional analgesia or TAP block [1, 5, 17–19].

ITM when injected alone in patients having major surgery under GA advocates intra- and postoperative opioid-sparing effects. However, monitoring of RD in a high-dependency PACU is a major concern, especially in resource-limited settings (RLS) [6–9]. Although there is no consensus regarding the optimal dose of ITM for pain control in the 24 h postsurgery*,* there was a trend toward using ‘‘mini-doses’’ of ITM to reduce the risk of RD and maintain adequate extended analgesia [8, 20]. Dose–response studies suggested that ITM has a ceiling analgesic efficacy above which the risk of adverse events exceeds the benefits of improved pain control [20].

Despite the conflicting conclusions of dose–response of different ITM doses for PO pain management [21–23], our results in this study showed similarity with previous studies as 0.3 mg ITM was the ‘analgesic ceiling’ and the optimal dose for PO pain relief with the least side effects [21, 22]. In contrast, Hein et al. reported that no additional benefits for PO analgesia was recorded from increasing the dose over 0.2 mg ITM added to bupivacaine spinal anesthesia [23], which could be attributed to the additive effect and better analgesia achieved by the intrathecal combination of the local anesthetic and morphine targeting different sites of action [11].

Previous studies [24, 25] are consistent with our results regarding superior analgesia, hemodynamic stability and stress response attenuation of both ESPB and ITM for PO pain control. The research team adopted the use of lower blood glucose levels and serum cortisol levels as inexpensive surgical stress markers to express the stress response attenuation of both ESPB and ITM for PO pain management [25].

In the current study, the PO recovery parameters showed significantly longer time periods in the control group, which could be attributed to the adequate PO pain control in the intervention groups**.** Despite the conflicting conclusions of whether ESPB [5, 17, 19, 26] and ITM [8, 9, 23] are valuable to speed up recovery after surgery, both of them were effectively implemented in multimodal analgesic approaches to get a longer duration of PO pain relief, delay the time to first rescue analgesic request and reduce overall patients` PO opioid consumption [25, 26].

One of the postulated mechanisms of PO pruritus after ITM administration is the action of morphine's cephalad spread on the µ-opioid receptors and 5-HT_3_ receptors present in the dorsal horn of the spinal cord and in the spinal trigeminal nucleus located in the medulla (itch center) [27–31]. Furthermore, ITM-induced pruritus is a dose-related adverse effect [27–31] and the serum serotonin levels significantly increased after ITM injection [27]. In addition, ITM can induce pruritus due to stimulation of 5-HT_3_ receptors present in the dorsal horn of the spinal cord and medulla [27–31]. Compared to both the ESPB and control groups in the current study, the ITM group had higher incidences of nausea and pruritus 24 h after surgery and the investigators found significant differences between the ESPB and control groups regarding the incidence of nausea**.** The use of tramadol as a rescue analgesic for PO pain control is associated with increased incidence of PONV [32]. The results of previous studies [28–31] are in concordance with our recent findings regarding the pre-emptive intake of 3 mg IV granisetron (5-HT_3_ receptor antagonist), which resulted in a significant reduction in the severity of pruritus and the need for antipruritic treatment, but without reducing the number of patients experiencing PO pruritus in ITM group. The use of 5-HT_3_ receptor antagonists with a longer half-life extends beyond the peak of ITM-induced pruritus due to increased modulation of the serotonergic pathways [27]. The elimination half-life of granisetron is 8.95 h, with a duration of more than 24 h and its optimal dose for the prevention of PONV is 2 mg via an IV route [33].

The ITM administration for PO analgesia is associated with a frequent incidence of PONV [7–9, 21–23, 29–31], and previous studies for the treatment of PONV have proposed the 5-HT_3_ receptor interaction by opioids as a potential mechanism [29–31]. Also, the frequent use of rescue analgesic (tramadol) in the control group in this clinical study increased the incidence of PO nausea. Such a scenario made the use of a prophylactic dose of 3 mg IV granisetron [30–33] to reduce the incidence of PONV, an attractive option adopted by the investigators of this study.

Our results regarding the significance of PO sedation in both the ITM and ESPB groups compared to the control group could be attributed to the efficient PO analgesia in the intervention groups that reduced anxiety and enhanced sleep quality [34]. Because of fear of RD, anesthesiologists are hesitant to administer ITM [8]. Early RD (within 2 h of injection) due to ITM administration has never been recorded [35]. However, ITM administration was involved in all reports of clinically relevant delayed RD (6–12 h following injection) [8, 22, 35]. The heterogeneity in definition of ITM-induced RD limits the widespread use of ITM for PO pain control [8]. As a result of a dose-dependent relationship between the risk of RD and the dose of ITM, a ceiling dose of ≤ 0.3 mg ITM was recommended [9]. Current approaches have been implemented in monitoring and early recognition of patients who develop opioid-induced RD as a potentially avoidable cause of death and brain damage following surgery [36].

The research team`s results proved that prophylactic administration of 3 mg intravenous granisetron to patients receiving 0.3 mg ITM provided extended analgesia up to 24 h postoperatively with low burden of adverse drug reactions (28–31) in patients undergoing total abdominal hysterectomy under general anesthesia as compared to ESPB. Despite that the ITM group had higher incidences of nausea (*P* < 0.001) and pruritus (*P* < 0.001) 24 h after surgery compared to the ESPB group, the severity of nausea was comparable between the two groups (*P* > 0*.*05) (Table 2). Compared to ESPB, most of the pruritus cases in the ITM group were of a the mild form (*P* < 0.001) (Table 2) requiring no rescue antipruritic drug (*P* > *0.05*) (Table 2).

This study had several merits. First, the investigators demonstrated an efficient way to prepare the dose of ITM morphine with a single dilution method. Second, the ITM dose was adequate in providing PO analgesia without serious side effects (especially RD).Third, the investigators did not add ITM to the local anesthetic, which may potentiate the analgesic effects of ITM and avoid hemodynamic disruption. Fourth, this study had a control group with systemic analgesia alone, which clarified the true analgesic efficiency of both ESPB and ITM. Fifth, this study identified the benefits related to PO recovery in the ESPB and ITM groups.

## Limitations

First, this was a single center study. Therefore, multicenter research studies are required to enhance the reproducibility and generalizability of the results of this study. However, this limitation can be disregarded because Ain-Shams University Maternity Hospital is considered as one of the largest tertiary referral hospitals in Egypt. Second, cost-effectiveness analysis of the study medications and hospital LOS should be addressed in future clinical trials, especially in RLS.

A focus on diversity of analgesic requirements and PO pain control protocols following TAH will ameliorate patient safety, healthcare quality and PO outcomes, especially in RLS [37]. It is important to adopt improvements for PO pain techniques to identify inefficiencies, ineffective care, preventable errors and differences in anesthesia provider education and experience. ITM is a simple technique for extended PO pain relief [8, 9, 22, 38] and it does not require additional training or expensive equipment such as ultrasound machines [38].

## Conclusion

0.3 mg intrathecal morphine was superior to erector spinae plane block for postoperative pain relief, 24 h after surgery, regarding attenuated stress response, lower pain scores at rest and on coughing and lower tramadol consumption.

## Data Availability

Data are available from the authors upon reasonable request and with permission of Ain-Shams University Maternity Hospital.
